# Genetic analysis of grapevine root system architecture and loci associated gene networks

**DOI:** 10.3389/fpls.2022.1083374

**Published:** 2023-02-02

**Authors:** Dilmini Alahakoon, Anne Fennell

**Affiliations:** Agronomy, Horticulture, and Plant Science Department, South Dakota State University, Brookings, SD, United States

**Keywords:** LATERAL ORGAN BOUNDARY DOMAIN, protodermal factor, circadian rhythm, autophagy, grapevine, adventitious roots

## Abstract

Own-rooted grapevines and grapevine rootstocks are vegetatively propagated from cuttings and have an adventitious root system. Unraveling the genetic underpinnings of the adventitious root system architecture (RSA) is important for improving own-rooted and grafted grapevine sustainability for a changing climate. Grapevine RSA genetic analysis was conducted in an *Vitis sp.* ‘VRS-F2’ population. Nine root morphology, three total root system morphology, and two biomass traits that contribute to root anchorage and water and nutrient uptake were phenotyped. Quantitative trait loci (QTL) analysis was performed using a high density integrated GBS and rhAmpSeq genetic map. Thirty-one QTL were detected for eleven of the RSA traits (surface area, root volume, total root length, fresh weight, number of tips, forks or links, longest root and average root diameter, link length, and link surface area) revealing many small effects. Several QTL were colocated on chromosomes 1, 9, 13, 18, and 19. QTL with identical peak positions on chromosomes 1 or 13 were enriched for *AP2-EREBP*, *AS2*, *C2C2-CO*, *HMG*, and *MYB* transcription factors, and QTL on chromosomes 9 or 13 were enriched for the *ALFIN-LIKE* transcription factor and regulation of autophagy pathways. QTL modeling for individual root traits identified eight models explaining 13.2 to 31.8% of the phenotypic variation. ‘Seyval blanc’ was the grandparent contributing to the allele models that included a greater surface area, total root length, and branching (number of forks and links) traits promoting a greater root density. In contrast, *V. riparia* ‘Manitoba 37’ contributed the allele for greater average branch length (link length) and diameter, promoting a less dense elongated root system with thicker roots. *LATERAL ORGAN BOUNDARY DOMAIN (LBD or AS2/LOB)* and the *PROTODERMAL FACTOR (PFD2 and ANL2)* were identified as important candidate genes in the enriched pathways underlying the hotspots for grapevine adventitious RSA. The combined QTL hotspot and trait modeling identified transcription factors, cell cycle and circadian rhythm genes with a known role in root cell and epidermal layer differentiation, lateral root development and cortex thickness. These genes are candidates for tailoring grapevine root system texture, density and length in breeding programs.

## 1 Introduction

Grapevine (*Vitis* sp.) is one of the most economically important fruit crops cultivated in the USA and the world (Nass, 2019). The increased temperature, water, and pest stresses associated with climate change frequently exceeds practical viticulture solutions. Therefore, to maintain an ecologically sound production system, development of cultivars with improved biotic and abiotic stress tolerance, water use, fruit quality, yield, and manageability is necessary (Limera et al., 2017). Grapevines are vegetatively propagated and either grown own-rooted or grafted to a rootstock. Adventitious roots provide anchoring and mechanical support, absorb water and nutrients from the soil, store carbohydrates, and exposure to beneficial soil microorganisms (Bellini et al., 2014). They serve as the only interface to sense and respond to changing soil environments, enabling plants to overcome abiotic stress challenges. The grape phylloxera epidemic of the late 1800s in Europe spurred the development of phylloxera resistant rootstocks and hybrid own-rooted grapevines. A large portion of the resulting rootstock cultivars are closely related and selected for phylloxera resistance and graft compatibility rather than stress tolerance and there has been limited genetic analysis of grapevine root systems (Walker et al., 2014; Ollat et al., 2016; Dalbó and Souza, 2019; Riaz et al., 2019). In contrast, genetic analysis of grapevine scion cultivars for improved fruit quality, abiotic and biotic resistance, cold tolerance, seedlessness, and other enological and phenological traits have proceeded rapidly (Correa et al., 2014; Delrot et al., 2020; Gautier et al., 2020; Zinelabidine et al., 2021; Vezzulli et al., 2022). The need for improved stress tolerance and pest resistance in changing climatic conditions highlights the need for a greater understanding the genetic basis of root system architecture (RSA) to continue development of improved own-rooted cultivars and rootstocks (Serra et al., 2014; Hugalde et al., 2021b).

The RSA is the spatial distribution of roots and reflects the shape, three-dimensional distribution, and branching pattern of post-embryonically generated roots or adventitious roots (Lynch, 1995; Satbhai et al., 2015). Root architecture can be described by morphology, topology, geometry, and growth dynamics (Lynch, 1995). Morphology refers to root shape, diameter, length, and orientation. The topology is the connection of roots through branching such as primary or secondary order roots. The positional gradient of roots or root biomass/length and their soil depth contribute to root geometry. Root growth rate and lateral emergence rate are examples of root dynamics. Roots have a seasonal growth pattern that optimizes nutrient and water uptake and anchorage. The main environmental factor that impacts root system growth and development is the heterogeneity of the soil properties such as bulk density, texture, water availability, and nutrient content (Smart et al., 2006; Comas et al., 2013; Serra et al., 2014; Yildirim et al., 2018). RSA shows considerable variation among species, genotypes within a species, and cultivars (Lynch, 1995). Root trait mapping studies for the annual crops rice (Uga et al., 2013), soybean (Valliyodan et al., 2017), barley (Robinson et al., 2018), and wheat (Zhang et al., 2019) have identified colocated quantitative trait loci (QTL) for root branching, morphology, biomass, and yield. In the deciduous tree *Populus*, adventitious root QTL are colocated with related shoot traits indicating the importance of the root system on shoot development (Han et al., 1994; Sun et al., 2019). Scion transpiration rate and acclimation to water deficit are controlled by rootstock genetics (Marguerit et al., 2012). An interspecific *V. vinifera* L. × *V. riparia* Michx. rootstock population study indicates that scion transpiration is controlled by a small number of loci each responsible for<10% of the phenotypic variance and suggests that hormonal (abscisic acid) and hydraulic (aquaporins) signaling genes play a role in the rootstock genotype response to water deficit (Marguerit et al., 2012). Studies of *V. riparia* × *V. labrusca* and ‘Riparia Glorie’ drought sensitive rootstocks have shallower root distribution than the drought-tolerant rootstocks ‘110R’ (*Vitis berlandieri* × *Vitis rupestris*) and ‘Ramsey’ (*Vitis champinii*) (Fort et al., 2017). The interspecific rootstocks *V. riparia* × *V. rupestris* Scheele. and *V. vinifera × V. berlandieri* Planch. had different capacity to withstand water and sodium chloride stress indicating that genotype variation contributes to root physiological and functional differences in response to the environment (Meggio et al., 2014). Petiolar nutrient concentration is influenced by rootstock and *V. riparia* contributes to lower petiolar magnesium and phosphorus and higher sulphur concentration in the scions in comparison to rootstocks with *V. rupestris* or *V. berlandieri* (Gautier et al., 2020).

The adventitious root system and rootstocks contribute to the sustainability of the grapevine; however, most reported root traits are controlled by multiple genes, each governing small effects and often changing with environmental conditions (De Dorlodot et al, 2007; Cooper et al., 2009). Marker assisted selection for efficient grapevine root systems has been limited due to lack of rapid and accurate phenotyping methods for roots and linkage of root phenotype to crop productivity (Lynch, 1995). The degree of diversity and associated variation in root traits, their complex genetic control, and the strong environmental effect on morphological traits inhibits traditional genetic studies in grapevine root systems (De Dorlodot et al, 2007). These difficulties result in a gap in our understanding of the genetic control of RSA and contribute to the lack of markers needed to assist in selection for improved root systems in own-rooted or rootstock cultivars. Therefore, this study was undertaken to explore the genetic architecture of root morphology in an interspecific F_2_ grapevine population and identify loci and candidate gene influencing adventitious root system morphology.

## 2 Materials and methods

### 2.1 Plant material and growing conditions

The VRS-F_2_ diploid population was produced by selfing a single F_1_ (*V.* sp. ‘16_9_2’) developed from a cross between *V. riparia* Michx. (seed parent, ‘Manitoba 37’, PI#588289) and *V.* sp. ‘Seyval blanc’ (pollen parent, VIVC#11558) (Fennell et al., 2005). Six-year-old potted VRS-F_2_ vines for this study were cycled annually through the greenhouse and cold storage in South Dakota State University, Brookings, SD (44.31°C N, 96.80°C W). The ecodormant vines were root pruned and repotted in soil, perlite, and peat growing medium (1:2:2 by volume), grown five months, induced into dormancy by natural short daylengths, and returned to cold storage after harvesting canes. Dormant replicate canes were collected from 266 F_2_ individuals, the population parent, and grandparents in early November and stored at 4°C as three-node cuttings (nodes 3-5 from the cane base) keeping genotype identity. For this study, chilling fulfilled canes were placed in a container with 10 cm water layer for three days to ensure uniform hydration. After three days of hydration, single node cuttings with swollen buds (six cm cane sections) were selected from the center of the 3-node cutting and placed randomly in a rooting box (60×45×15 cm (length x width x depth)) of perlite. Six replicate cuttings were used for each VRS-F_2_ genotype, 28 replicates for the F1 parent, and 18 replicates for each grandparent. Cuttings were placed randomly (maintaining identity) 7.5 cm apart within row and between row spacing. The root boxes were flooded and drained daily to maintain uniform moisture content. The rooting study was conducted in the greenhouse with >14-hour natural daylength at 26 ± 3°C and 80% relative humidity.

### 2.2 Trait measurements

After 35 days, plants were harvested and cleaned using tap water to remove all perlite particles. Samples were stored (in plastic bags with 1 ml of water) in a 4°C cooler until they were scanned. Genotypes and their RSA replicate-identity were maintained throughout the experiment. Each root system was scanned using Epson scanner (PERFECTION V700PHOTO, Seiko Epson Corporation, Tokyo, Japan). Each root system was scanned twice (two different faces on scanner) to get the mean traits. After scanning, the length of the longest root was measured with a ruler. All the roots were then cut off at the collar region. The root fresh weight (FW) was measured, and the roots were dried at 60°C for 48 hours and the dry weight (DW) measured. Finally, the diameter (CD) and length (CL) of the single node cane section were measured manually. Eleven traits related to RSA were measured using WinRhizo software Reg 2016a (Regent Instruments Inc, Quebec, Canada). The root and propagule traits were categorized into four groups (total root system characters, individual root characters, average root characters, and stem characters) based on their morphology (**Table 1**). These root trait category names and trait name abbreviations are used throughout the results and discussion.

**Table 1:** Root system architecture trait abbreviations and measurement unit.

Trait	Abbreviation	Unit	Description
Total root system traits			
Surface area	SA	cm^2^	Whole root system surface area
Root volume	RV	cm^3^	Whole root system volume
Total root length	RL	cm	Whole root system length
Fresh weight	FW	g	Whole root system weight after harvesting
Dry weight	DW	g	Whole root system weight after keeping 48 hours at 60 ^0^C
Individual root traits			
Number of tips	NT	Count	Root tip number of or lateral root number. These lateral roots include primary, secondary, and tertiary laterals
Number of forks	NF	Count	Fork number or divides in whole root system
Number of links	NL	Count	Root system connectivity or piece of root between two branching points (interior link) or between a branch and a meristem (exterior link).
Longest root	LR	cm	The longest root length
Average root traits			
Average diameter	AD	mm	Average diameter of all primary, secondary, and tertiary root links
Average link length	ALL	cm	Average length of a connection or distance between branches
Average link surface area	ALSA	cm^2^	Average link surface area
Average link diameter	ALD	mm	Average link diameter
Average link branching angle	ALBA	degree	Average angle between two links or lateral root angle from its parent root
Cane propagation section traits			
Cane section diameter	CD	mm	Single node cane cutting diameter at collar region
Cane section length	CL	cm	Single node cane cutting length

### 2.3 Statistical analysis

For each trait, the mean, median, minimum, maximum, and standard error were calculated. The grandparent’s trait means were tested for significant differences using a t-test in R (R Core Team, 2019). Only VRS- F_2_ genotypes (239) with no missing replicates were used for analysis. The genotype mean trait values for 16 traits were explored for their correlation, major trait contribution by principal component (PCA), and genotype relationship to grandparents or parent by cluster analysis. The data were analyzed using different packages in R statistical software (R Core Team, 2019). Descriptive data analyses were performed using *dplyr* (Wickham et al., 2019) and *psych* (Revelle, 2018) packages. The *Hmisc* package (Harrell, 2019) was used to calculate significant trait Pearson correlation coefficients. Principal component analysis was conducted for 16 traits (including 14 RSA traits and 2 stem traits) to identify trait contribution to RSA using the *factoextra* package (Kassambara and Mundt, 2017). Unsupervised k-means clustering method was used to categorize the 239 VRS-F_2_ genotypes with zero missing trait values using *cluster* package (Maechler et al., 2019).

### 2.4 Integrated VRS-F_2_ map

An integrated VRS-F_2_ GBS-rhAmpSeq genetic map using 1449 GBS markers as described in Yang et al. (2016) and 1970 rhAmpSeq markers developed from a genus-wide core genome and described in Zou et al. (2020). Genotyping and SNP calling of GBS markers were performed as described in Yang et al. (2016). rhAmpSeq marker development including DNA processing and genome assembly, core-genome construction, genus-wide variant calling, marker design pipeline, rhAmpSeq sequencing and genotyping, and quality control were detailed in Zou et al. (2020). As described in Alahakoon et al. (2022) distorted GBS and rhAmpSeq markers were tested using a threshold 1 × 10^-21^ chi-square p-value. Linkage groups containing a total of 2519 markers across 19 chromosomes with LOD = 5 were established using JoinMap (version 5, Kyazma B. V., Wageningen, Netherlands) (Alahakoon et al., 2022). Quantitative mapping was carried out for each of the 14 root traits, using the VRS-F_2_ GBS-rhAmpSeq integrated genetic map and *R/qtl* software (Broman et al., 2003; Alahakoon et al., 2022). Normal distribution of RSA phenotypes was measured using Shapiro-Wilk test and those not normally distributed were transformed to achieve normality. QTL were determined using *scanone* function in R/qtl. A permutation test was performed to identify 5% genome-wide log_10_ likelihood ratio (LOD) threshold (1,000 permutations). Eight traits had multiple significant QTL and QTL modeling was performed for those eight traits. All significant QTL used to build an additive model (*y*~*x*_*1*_+⋯+*x*_*n*_+*∈*, where ***y*** is the trait, ***x*** is the QTL, ***n*** is the number of significant QTL, and ***∈*** is the error term). Modeling of multiple QTL for a given trait was conducted according to the R/qtl package and QTL contributing to the model were tested for significant interactions and no interactions were significant. The QTL peak position marker, LOD score, percent of variation explained by individual and modeled QTL were evaluated by analysis of variance. Using Bayesian method, 95% confidence intervals were calculated. Dominant allele and the contributing grandparent for each of the modeled trait were identified.

### 2.5 Pathway analysis

Pathway enrichment analysis of individual traits was conducted by extracting the genes in the *Vitis vinifera* ‘PN40024 12X.v2 genome using 700 Kb either side of peak position considering that candidate genes should be within 3-4 cM of peak position or 1.4 Mb total based on grape genome size (Cipriani et al., 2011; Hugalde et al., 2021a). This 1.4 Mb region was used to identify enriched VitisNet pathways and candidate genes (Cipriani et al., 2011; Grimplet et al., 2012). The pathways were analyzed using the VitisNet functional annotation of the *Vitis vinifera* ‘PN40024 12X.v2’ genome and Fisher’s test (*p*-value< 0.05 for enrichment (Grimplet et al., 2012; Osier, 2016). VitisNet pathway enrichment analysis was also conducted for QTL that had three or more traits with the same peak position to identify RSA candidate genes.

## 3 Results

### 3.1 RSA phenotypic variation among grandparents, the parent and F_2_ population

**Figure 1:**
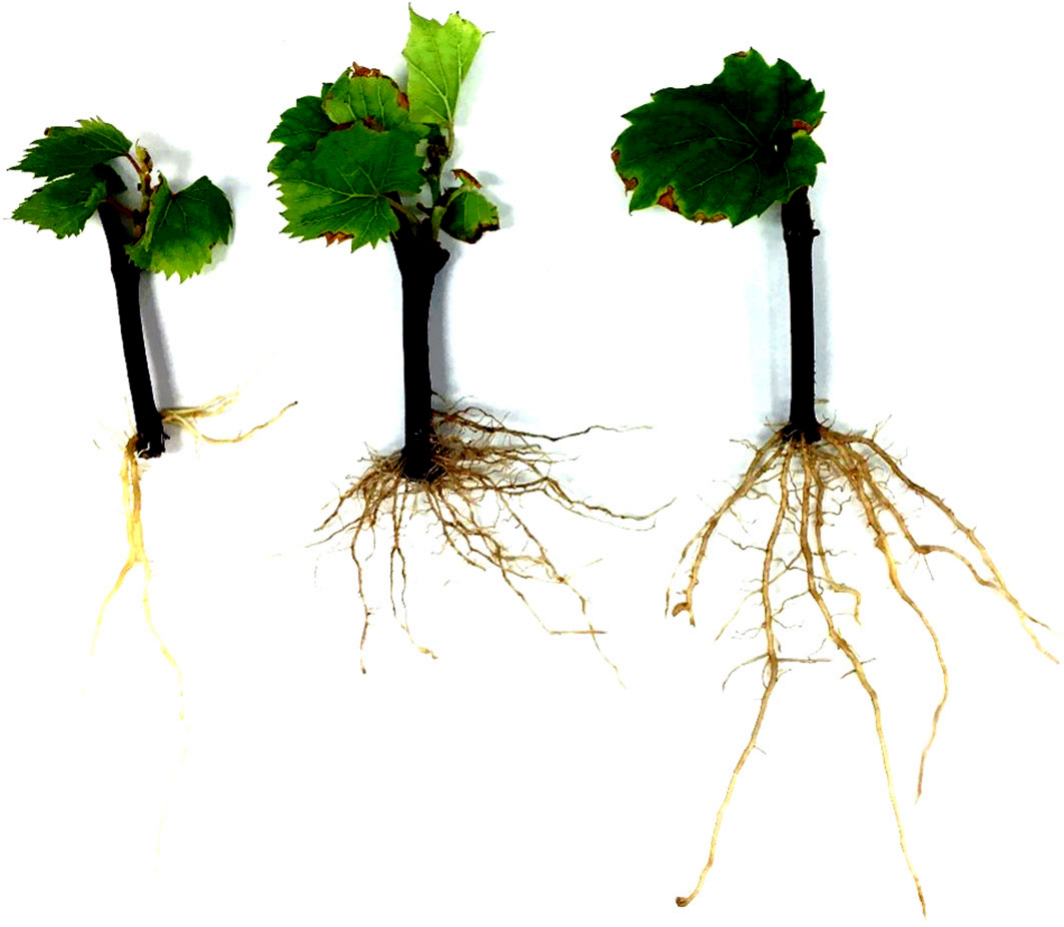
Representative adventitious root systems for the grandparents and parent of the VRS-F_2_ population. *V. riparia* ‘Manitoba 37’ (left) and ‘Seyval blanc’ (middle), and the VRS-F_2_ population parent 16-9-2 (F_1)_ (right).

Grapevine adventitious roots that developed under well-watered conditions were analyzed for 239 VRS-F_2_ genotypes. The RSA phenotypic variation for the grandparents and the parent of the VRS-F_2_ population is shown in **Figure 1**. Significant differences between means of the grandparents were detected for all measured RSA traits except RV, FW, LR, and ALBA (**Table 2**). SA, TRL, DW, NT, NF, and NL values were greater for ‘Seyval blanc’ than for *V. riparia* ‘Manitoba 37’ and AD, ALL, ALSA, and ALD were greater for *V. riparia* than for ‘Seyval blanc’. The values for the VRS-F_2_ population showed wide variation for all traits with values ranging from less to greater than the means of the parent and grandparents (**Table 2**). Original trait distributions showed deviation from the normality (**Supplementary Figure 1**). Traits were normalized and a Shapiro-Wilk normality test *p*-values revealed that eleven traits were successfully transformed with *p*-value > 0.05.

**Table 2:** Descriptive RSA trait statistics for *V. riparia* ‘Manitoba 37’ and ‘Seyval blanc’ (grandparents), F_1_ (parent), and the F_2_ population.

Trait	VR37	Seyval	t test	F_1_	VRS-F_2_	Min	Max
Total root system traits							
SA	15.4 ± 1.5	21.0 ± 1.1	*	44.4 ± 2.5	28.7 ± 0.4	1.7	99.4
RV	0.4 ± 0	0.4 ± 0	NS	0.8 ± 0.1	0.6 ± 0	0	2.3
TRL	50.7 ± 7.6	103.0 ± 5.6	*	199.6 ± 11.9	114.1 ± 1.6	3.8	403.8
FW	0.5 ± 0.1	0.5 ± 0	NS	1.0 ± 0.1	0.7 ± 0	0	3.8
DW	0 ± 0	0.1 ± 0	*	0.1 ± 0	0.1 ± 0	0	0.3
Individual root traits							
NT	171.7 ± 25.4	387.2 ± 24.0	*	564.2 ± 32.8	340.8 ± 4.7	13.0	1528.0
NF	87.7 ± 12.1	234.1 ± 9.2	*	554.9 ± 47.3	284.4 ± 4.6	2.0	1274.0
NL	218.2 ± 28.8	547.9 ± 21.4	*	1128.0 ± 84.4	615.1 ± 9.2	14.0	2381.0
LR	9.5 ± 1.4	11.7 ± 0.6	NS	13.7 ± 0.7	11.5 ± 0.1	1.0	33.7
Average root traits							
AD	1.1 ± 0.1	0.7 ± 0	*	0.7 ± 0	0.8 ± 0	0.5	1.7
ALL	0.3 ± 0	0.2 ± 0	*	0.2 ± 0	0.2 ± 0	0.1	1.2
ALSA	0.1 ± 0	0 ± 0	*	0 ± 0	0.1 ± 0	0	1.2
ALD	0.8 ± 0.1	0.6 ± 0	*	0.7 ± 0	0.7 ± 0	0.1	1.6
ALBA	41.4 ± 0.4	41.0 ± 0.4	NS	42.0 ± 0.1	41.6 ± 0	31.0	49.7
Propagation cane section traits							
CD	4.5 ± 0.2	6.7 ± 0.2	*	6.5 ± 0.2	5.8 ± 0	3.0	13.0
CL	6.8 ± 0.2	5.7 ± 0.1	*	6.0 ± 0	6.1 ± 0	2.7	10.7

Values are mean ± standard error (se); VR37, *V. riparia* ‘Manitoba 37’, n=18; ‘Seyval blanc’, n=18; F_1_, the parent, n=28; VRS-F_2_, n=239; Min, observed minimum value in the study; Max, observed maximum value in the study; *, statistically significant at p-value ≤ 0.05. NS, not significant at p-value ≤ 0.05.

### 3.2 Trait correlations

The total root system traits and individual root characteristics traits showed significant (*p*-value<0.05) and strong positive correlations (**Table 3**). Average root characteristic traits did not display a strong correlation with total root system traits or individual root characteristics traits. The AD and total root system characteristics and individual root characteristics traits that would contribute to root length and number had a negative correlation. AD had a positive relationship with the other average traits (ALL, ALSA, and ALD). The cane propagule section diameter and length (CD and CL) did not show a strong correlation with any RSA traits.

**Table 3:** Pearson correlation coefficients for RSA traits in VRS-F_2._.

	Total Root System Traits						Individual Root Traits				Average Root Traits					Cane Trait
	SA	RV	TRL	FW	DW	NT	NF	NL	LR	AD	ALL	ALSA	ALD	ALBA	CD	CL
**SA**	1.0	0.0	0.0	0.0	0.0	0.0	0.0	0.0	0.0	0.0	0.0	0.0	0.1	0.0	0.0	0.0
**RV**	**0.9**	1.0	0.0	0.0	0.0	0.0	0.0	0.0	0.0	0.0	0.0	0.0	0.0	0.0	0.0	0.0
**TRL**	**0.9**	**0.8**	1.0	0.0	0.0	0.0	0.0	0.0	0.0	0.0	0.0	0.0	0.0	0.0	0.0	0.0
**FW**	**0.9**	**0.8**	**0.8**	1.0	0.0	0.0	0.0	0.0	0.0	0.3	0.2	0.7	0.0	0.0	0.0	0.0
**DW**	**0.9**	**0.8**	**0.8**	**0.8**	1.0	0.0	0.0	0.0	0.0	0.3	0.0	0.2	0.0	0.0	0.0	0.0
**NT**	**0.8**	**0.6**	**0.8**	**0.7**	**0.6**	1.0	0.0	0.0	0.0	0.0	0.0	0.0	0.0	0.0	0.0	0.0
**NF**	**0.8**	**0.7**	**0.9**	**0.7**	**0.7**	**0.7**	1.0	0.0	0.0	0.0	0.0	0.0	0.0	0.0	0.1	0.0
**NL**	**0.9**	**0.7**	**0.9**	**0.7**	**0.7**	**0.8**	**1.0**	1.0	0.0	0.0	0.0	0.0	0.0	0.0	0.3	0.0
**LR**	**0.7**	**0.6**	**0.7**	**0.6**	**0.6**	**0.7**	**0.4**	**0.5**	1.0	0.0	0.0	0.0	0.0	0.0	0.0	0.0
**AD**	**-0.1**	**0.2**	**-0.4**	0.0	0.0	**-0.4**	**-0.4**	**-0.4**	**-0.2**	1.0	0.0	0.0	0.0	0.1	0.0	0.0
**ALL**	**0.0**	**0.1**	**-0.1**	0.0	**0.1**	**-0.3**	**-0.3**	**-0.3**	**0.1**	**0.4**	1.0	0.0	0.0	0.0	0.0	0.0
**ALSA**	**-0.1**	**0.1**	**-0.2**	0.0	0.0	**-0.1**	**-0.2**	**-0.2**	**-0.1**	**0.4**	**0.4**	1.0	0.0	1.0	0.0	0.1
**ALD**	**0.0**	**0.3**	**-0.2**	**0.1**	**0.1**	**-0.4**	**-0.1**	**-0.1**	**-0.3**	**0.7**	**0.2**	**0.3**	1.0	0.9	0.0	0.7
**ALBA**	**-0.1**	**-0.1**	**-0.1**	**-0.1**	**-0.1**	**-0.1**	0.0	0.0	**-0.2**	0.0	**-0.2**	0.0	0.0	1.0	0.0	0.1
**CD**	**0.2**	**0.3**	**0.1**	**0.2**	**0.3**	**-0.1**	0.0	0.0	**0.1**	**0.3**	**0.2**	**0.1**	**0.3**	**-0.1**	1.0	0.0
**CL**	**0.2**	**0.2**	**0.1**	**0.2**	**0.1**	**0.2**	**0.1**	**0.1**	**0.1**	**0.1**	**-0.1**	0.0	0.0	0.0	**-0.1**	1.0

Pearson correlation coefficients in left column; significant correlation coefficients are in bold (p-value<0.05). Pearson correlation test p-values are across the top.

### 3.3 Morphological traits explain major part of the RSA

**Figure 2:**
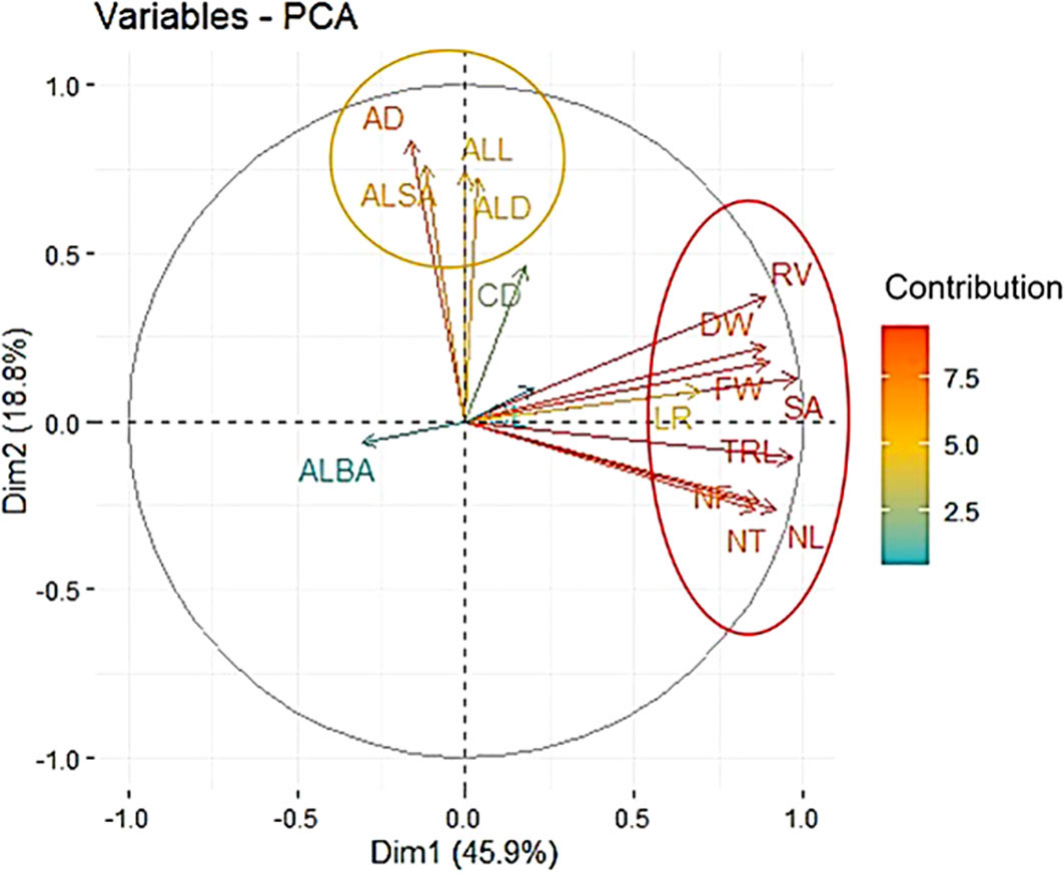
Root system architecture trait principal component biplot. The first (Dim1) and second (Dim2) principal component dimensions are in x and y-axis respectively. Red and orange ovals represent clusters formed. Vector color represents the percent contribution of each individual trait to the first and second principal components.

The principal component analysis was performed for all RSA traits measured to identify the trait contribution to RSA. The first principal component (Dim1), and second (Dim2), and third (Dim3) explained 45.9%, 18.8%, and 10.3% of the root system variation, respectively. The first dimension was predominately explained by total root system characteristics and individual root characteristics traits. Average root characteristics traits contributed to the second dimension. ALBA mainly characterized the third dimension. Traits that contributed to first and second dimensions clustered separately in the PCA biplot (**Figure 2**).

### 3.4 Genetic diversity in VRS-F_2_ population

**Figure 3:**
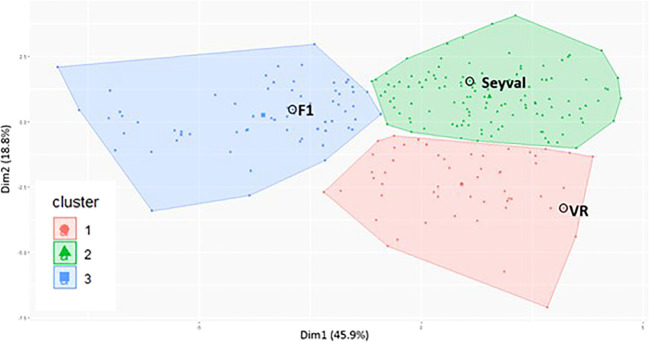
VRS-F2 population cluster plot for 14 root system traits and 2 propagule traits. Cluster plot was generated by using k-means clustering method and principal component analysis. VR indicates V. riparia ‘Manitoba 37’, Seyval indicates ‘Seyval blanc’ and F1 indicates the VRS-F2 population parent. Dots not circled represent VRS-F2 genotypes.

Cluster analysis of the 14 RSA traits and the 2 propagule traits classified 239 genotypes into three groups (**Figure 3**). Cluster 1 comprised 55 genotypes plus the female grandparent, *V. riparia* ‘Manitoba 37’. A second cluster contained ‘Seyval blanc’ and 120 genotypes, while the third cluster included 66 genotypes plus the F_1_ parent.

### 3.5 QTL detection and colocalization

**Figure 4:**
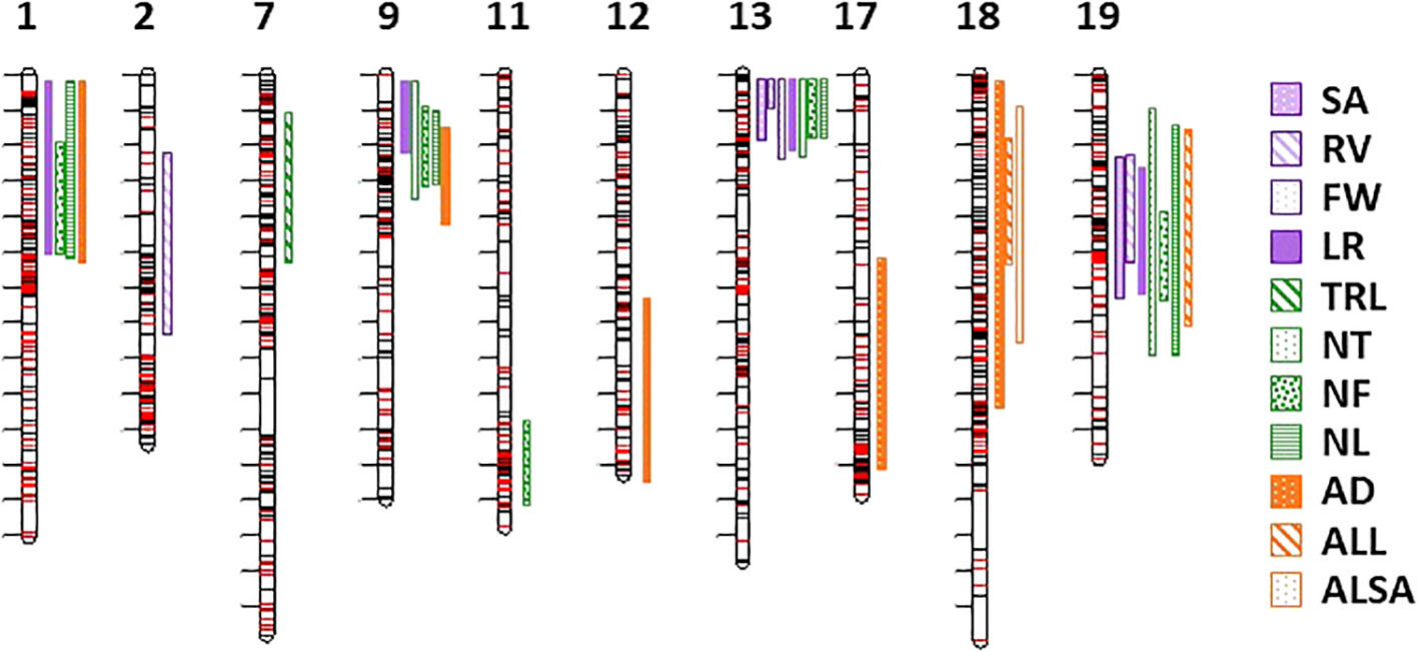
Chromosomal location of 31 QTL for eleven RSA traits. Chromosome number is at the top of the image. Legend on right of image identifies RSA traits with QTL (surface area (SA); root volume (RV); fresh weight (FW); total root length (TRL); longest root (LR); number of tips, forks, and links (NT, NF, NL); and average diameter, link length, and link surface area (AD, ALL, ALSA). Length of the band identifies the confidence interval.

Thirty-one QTL were detected for 11 RSA traits on chromosomes 1, 2, 7, 9, 11, 12, 13, 17, 18, and 19 (**Table 4**, **Figure 4**). No QTL were observed for the DW biomass trait, ALBA, and ALD average morphology traits or the CD and CL propagule traits. Multiple QTL were closely located on chromosomes 1, 9, 13, 18, and 19. In chromosomes 1, 9, 13, and 19 there were five identical peak positions for three or more trait QTL on chromosome 1 (NF, NL, and TRL; 3.01 Mb), 9 (TRL, NT, NF, and NL; 0.89 Mb), 13 (SA, RV, and TRL; 0.24 Mb) and (NT, NF, and NL; 0.51 Mb), and 19 (SA, TRL, NT, and NL; 5.92 Mb) (**Table 4**). The two separate peak positions for multiple traits on chromosome 13 were close to the end of the chromosome and had overlapping and small confidence intervals; therefore, for enrichment analysis they were analyzed as one position. None of the QTL identified on chromosome 18 had identical peak positions (**Table 4**). Single QTL were found on chromosomes 2 (RV), 7 (LR), 11 (NF), 12 (AD), and 17 (AD) (**Table 4**).

**Table 4:** Summarized QTL information for each trait.

Trait	Chr	LOD	Peak position (Mb)	Marker at the peak position	% variation explained by the QTL	Position (Mb) at 95% Bayesian interval
Total Root System Traits						
SA	13	5.1	0.24	GBS_13_242917	8.2	0.16:1.21
SA	19	3.8	5.92	GBS_19_5920951	6.1	3.41:10.51
RV	2	3.5	1.63	rh_2_1626662	5.2	0.95:4.83
RV	13	5.7	0.24	GBS_13_242917	8.7	0.16:0.94
RV	19	4.1	3.86	GBS_19_3856353	6.3	3.29:9.15
TRL	1	3.7	3.01	rh_1_3008587	5.3	0.06:6.88
TRL	9	4.4	0.89	rh_9_893002	6.3	0.23:1.47
TRL	13	4.2	0.24	GBS_13_242917	5.9	0.16:1.69
TRL	19	4	5.92	GBS_19_5920951	5.6	3.65:10.51
FW	13	4.1	1.43	rh_13_1426203	7.5	0.16:2.34
Individual Root Traits						
NT	9	3.1	0.89	rh_9_893002	4.7	0.23:4.35
NT	13	4.3	0.51	rh_13_507206	6.7	0.16:2.34
NT	19	3.7	5.92	GBS_19_5920951	5.6	1.22:19.08
NF	1	5	3.01	rh_1_3008587	6.2	1.79:6.88
NF	9	5.8	0.89	rh_9_893002	7.4	0.3:1.31
NF	11	4.4	18.59	GBS_11_18591914	5.5	6.82:20.12
NF	13	4.8	0.51	rh_13_507206	6	0.16:1.69
NF	19	3.7	8.79	rh_19_8787547	4.6	5.81:10.51
NL	1	4.3	3.01	rh_1_3008587	6.1	0.06:7.38
NL	9	5.4	0.89	rh_9_893002	7.7	0.38:1.31
NL	13	4.2	0.51	rh_13_507206	5.9	0.16:1.69
NL	19	3.7	5.92	GBS_19_5920951	5.1	1.74:19.08
LR	7	4.5	1.48	rh_7_1482314	7.7	1.24:5.27
Average Root Traits						
AD	1	4.2	1.79	GBS_1_1785738	5.5	0.06:7.64
AD	9	4.5	3.74	GBS_9_3741936	6	0.89:5.38
AD	12	3.1	9.59	GBS_12_9587096	4.1	7.53:24.26
AD	17	4.7	5.89	rh_17_5893163	6.2	3.25:18.02
AD	18	3.5	2.45	GBS_18_2451088	4.6	0.23:14.49
ALL	18	4	5.91	GBS_18_5914731	6.7	2.44:7.62
ALL	19	4.2	7.46	GBS_19_7455111	7	1.74:17.62
ALSA	18	4	6.94	rh_18_6936362	7.1	1.49:10.91

chr, chromosome; LOD, likelihood ratio comparing the hypothesis of a QTL at a position versus that of no QTL % variation, the percent variation explained by each QTL; rh, rhAmpSeq marker; GBS, genotyping by sequencing marker.

### 3.6 Identical QTL peak positions identify inter-relationship of root traits

**Figure 5:**
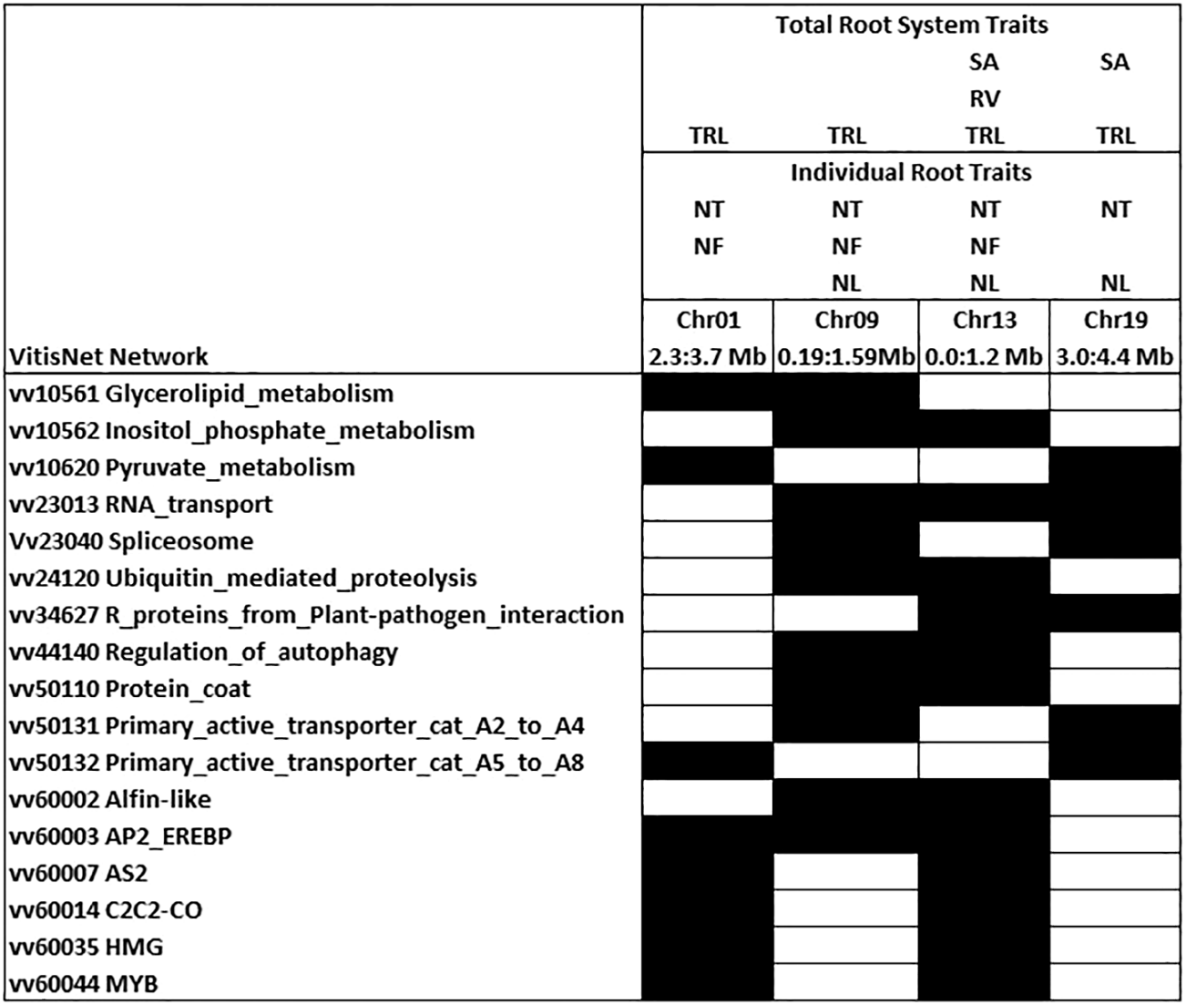
Enriched VitisNet networks for traits with identical QTL peak positions. Shading indicates the significantly enriched pathway (Fisher test p-value 0.05) in at least two of the loci. Trait abbreviations are noted in **Table 1**. All enriched pathways and contributing genes for each hotspot are identified in **Supplementary Table 1**. The two peak positions on chromosome 13 were merged into one hotspot as the 700 kb regions overlapped.

Chromosomes 1, 9, 13, and 19 had QTL peak positions that were identical for three to six root traits on each chromosome (**Figure 5**). These QTL had narrower confidence intervals than most of the single QTL. Examination of enriched VitisNet pathways underlying 700 kb either side of the peak position on each chromosome identified 17 enriched pathways that occurred in at least 2 of these loci and showed the interplay between individual and total root traits (**Figure 5**). All enriched pathways for each QTL are noted for the chromosome hotspot in **Supplementary Table 1**. In chromosomes 1 and 13 the enriched transcription factor pathway contained *LATERAL ORGAN BOUNDARIES DOMAIN* genes (*LBD4* and *ASYMETRIC LEAVES 2*, vv60007AS2) close to the QTL peak position. Enriched *ALFIN-LIKE* transcription factor and auxin signaling pathways on chromosome 9 contained an *ALFIN-LIKE PHD FINGER PROTEIN (ALFINDOM8*, vv60002ALFIN) and six auxin signaling genes near the QTL peak position. On chromosome 13, which had 6 traits mapping with an identical peak QTL, there were cell cycle and AS2 transcription factor pathways enriched and these included several cell cycle genes and two additional *LBD/LOB* genes in the loci. The QTL on chromosome 19 contained enriched actin cytoskeleton and *HOMEO BOX DOMAIN* (*HB*) transcription factor pathways which included *TORTIFOLIA1* (*TOR1*, which regulates cortical microtubules) and *PROTODERMAL FACTOR 2* (*PDF2*) near the peak position. On chromosome 1 and 19, circadian rhythm pathway was enriched with the presence of *CONSTANS-LIKE 11* and *16* and an *ALTERED RESPONSE TO GRAVITY* (*ARG1*) from the primary transporter enriched pathway were within the QTL confidence interval. Regulation of autophagy pathway was enriched (*AUTOPHAGY, APG 12* and *18*) for QTL on chromosome 9 and 13.

**Figure 6:**
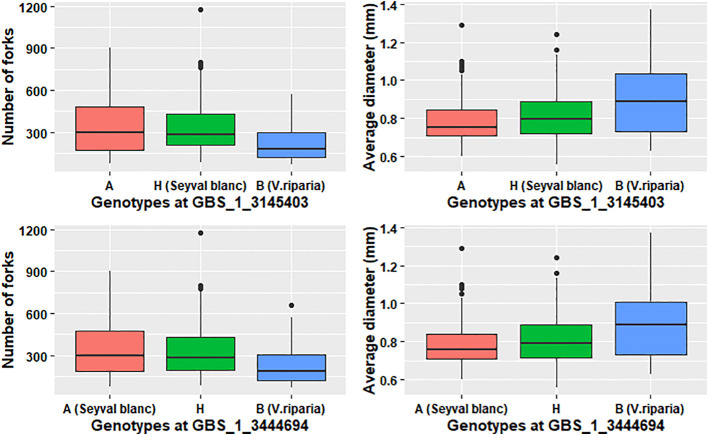
Genotype effect for markers surrounding *LATERAL ORGAN BOUNDARIES DOMAIN* candidate genes. The grandparent genotype is indicated in parentheses.

The variation explained by each QTL was small; therefore, models were tested for the individual root traits with more than one QTL. All the trait models were additive and no significant interactions were dectedted for this study. Modeling increased the explained phenotypic variation two- to five-fold for each trait to individual QTL (**Tables 4** and **5**). Both grandparents contributed dominant alleles for different RSA traits. ‘Seyval blanc’ grandparent contributed dominant allele for modeled traits (TRL, SA, RV, NT, NF, and NL). Three pathways were uniquely enriched for the individual NT, NF, and NL QTL; specifically, the regulation of autophagy (VV44140), the *APETALA2-ETHYLENE- RESPONSIVE ELEMENT BINDING PROTEIN* (VV60003 *AP2-EREBP*) and *ASYMMETRIC LEAVES2* (VV60007 AS2) transcriptions factors pathways (**Supplementary Table 1**). Exploration of the genotype effect for NF using two markers near the *LATERAL ORGAN BINDING DOMAIN* (*LBD*) candidate genes showed the dominant A allele in homozygous or heterozygous genotypes (A contributed by ‘Seyval blanc’) had a greater number of forks than the homozygous B genotypes (**Table 5**, **Figure 6**). A negative correlation existed between the NF and AD traits. Examination of the genotype effect for the same markers relative to the AD phenotype indicated that the dominant allele B is associated with greater AD. *V. riparia* contributed dominant allele for modeled traits AD and ALL (**Table 5**). The dominant allele contributor for the single QTL traits FW and LR was ‘Seyval blanc’ and for the ALSA trait it was *V. riparia*.

**Table 5:** RSA trait model phenotypic variation and genotype dominant allele contribution.

Trait	Chromosome location of QTL in model	Model LOD score	% Phenotypic variation	Dominant allele contribution
Total root system traits				
SA	13, 19	8.0	13.2	‘Seyval blanc’
RV	2,13,19	11.6	18.5	‘Seyval blanc’
TRL	1, 9,13,19	14.8	23.0	‘Seyval blanc’
Individual root traits				
NT	9, 13, 19	10.1	16.4	‘Seyval blanc’
NF	1, 9, 11, 13, 19	21.7	31.8	‘Seyval blanc’
NL	1, 9, 13, 19	16.2	25.5	‘Seyval blanc’
Average root traits				
AD	1, 9, 12, 17, 18	18.4	17.7	*V. riparia* ‘Manitoba 37’
ALL	18, 19	7.8	13.3	*V. riparia* ‘Manitoba 37’

### 3.7 VitisNet Pathway enrichment analysis in modeled traits

**Figure 7:**
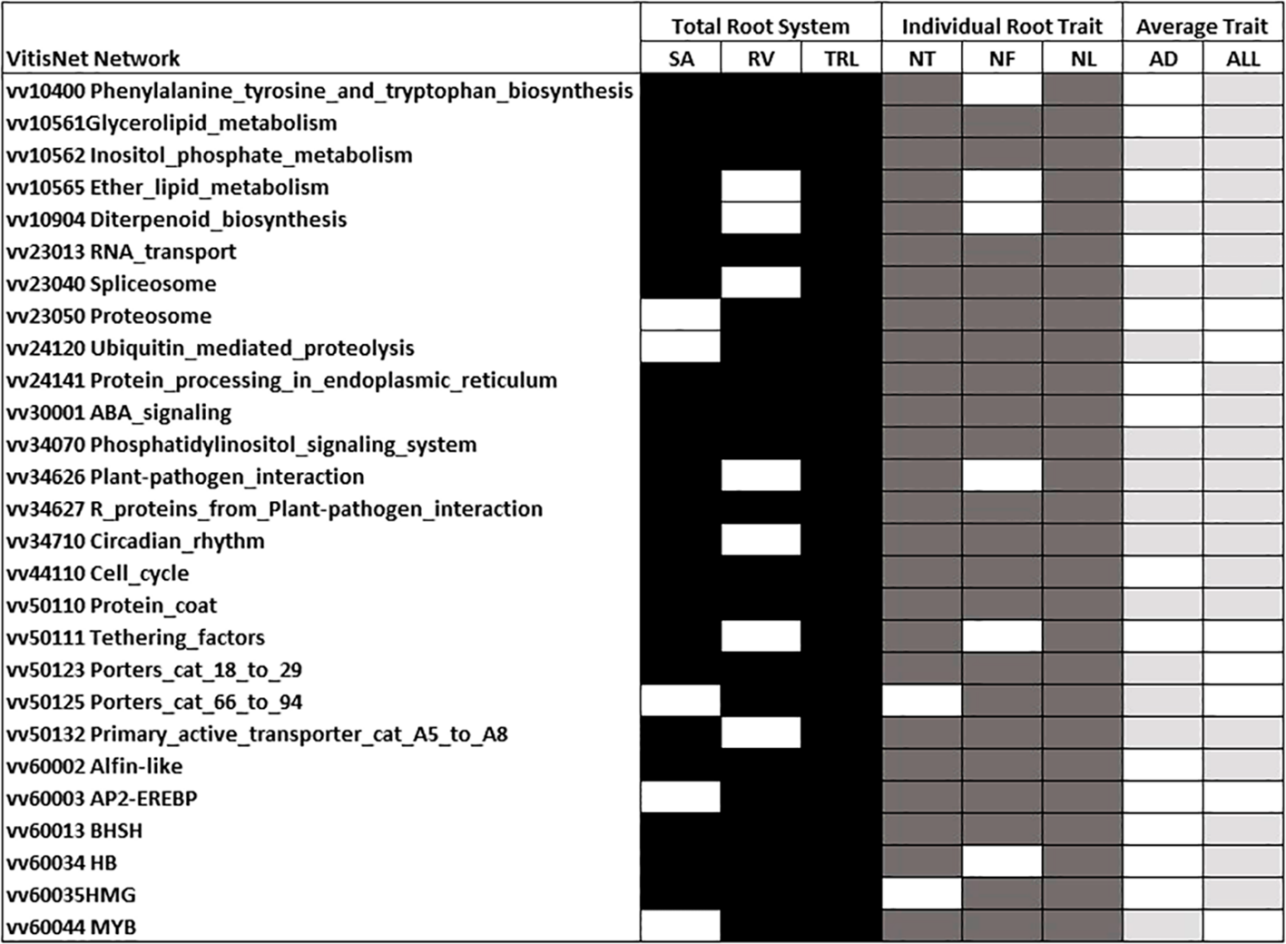
Enriched VitisNet pathways for modeled total root system, individual root and average traits. Shading indicates the significantly enriched pathway for total root system (black), individual root (dark gray), and average root (light gray/) traits (Fisher test p-value 0.05) White indicates not enriched. Only pathways enriched in at least five of the modeled traits are presented. All enriched pathways for an individual modeled trait are in **Supplementary Table 2**. Trait abbreviations are noted in **Table 1**.

An analysis of all genes underlying the 700 Kb region either side of each QTL peak position for modeled traits indicated > 2200 unique genes for the regions underlying modeled trait QTL. Enriched VitisNet pathways and their contributing genes for all modeled trait QTL are indicated in **Supplementary Table 2**. The number of enriched pathways varied by trait and there were 27 pathways enriched for five or more of the eight modeled traits (**Figure 7**). TRL and individual root traits (NT, NF, and NL) showed the most enriched pathways in-common (**Figure 7**), similarly they had the greatest co-localization of QTL (**Table 4**). The individual root traits (NT, NF, and NL) showed 25 in-common enriched pathways underlying their QTL, with ABA and Auxin signaling, circadian rhythm, cell cycle, Regulation of autophagy, *AP2_EREBP* and *BASIC HELIX-SPAN-LOOP-HELIX* BHSH transcription factors (**Supplementary Table 2**). The modeled average traits AD and ALL had 12 enriched pathways in common and nine of these were in common with other root traits (**Supplementary Table 2**; **Figure 7**). Of the average root traits, ALL had the most enriched pathways in common with the individual root traits and TRL; notably circadian rhythm, cell cycle and *HB* and *MYB* transcriptions factors (**Supplementary Table 2**).

## 4 Discussion

### 4.1 VRS-F_2_ grandparents differ in trait contribution to RSA

The challenge of increased summer temperatures and decreased water availability associated with climate change emphasizes the need for improved own-rooted grapevine or rootstock cultivars to maintain globally sustainable production (Smart et al., 2006; Delrot et al., 2020; Schmitz et al., 2021). While significant effort is paid to the genetic analysis of cluster and canopy architecture, less information is available for the grapevine root architecture as this hidden portion of the plant is more difficult to phenotype (Iandolino et al., 2013; Correa et al., 2014; Li et al., 2019; Krzyzaniak et al., 2021; Zinelabidine et al., 2021). The highly heterozygous grapevines are vegetatively propagated as own-rooted or grafted on rootstock to maintain their unique traits; thus, the entire root system of grapevines in production is formed of adventitious roots (Smart et al., 2002). In typical vine propagation, the cane or stem size impacts the available carbon and vascular development for root development in cuttings (Smart et al., 2002). In this study there was a very low correlation between RSA and cutting size suggesting that the cutting size was uniform and resulted in negligible variation across the genotypes. For multigenic traits like root system architecture, selection of parents that show genetic and phenotypic divergence increases the power of QTL detection (Hung et al., 2012). In this study, a VRS-F_2_ mapping population known to segregate for cold hardiness and berry quality traits was used to explore the genetic characteristics of a young grapevine adventitious root system (Fennell et al., 2005; Yang et al., 2016; Fennell et al., 2019; Alahakoon et al., 2022). VRS-F_2_ progeny showed morphological variation across all traits and progeny clustered with the parent or one of the grandparents, indicating that genetic contribution from both grandparents were important in determining the RSA. The SA, TRL, NT, NF, NL, and LR traits provide measures of the total root system and are key factors influencing soil exploration (vertical and horizontal) and play a role in anchorage and water and mineral nutrient uptake (Comas et al., 2013; Yildirim et al., 2018). Exploration of the LBD candidate gene markers identified in QTL hotspot showed that the grandparent ‘Seyval blanc’ contributed the dominant allele for the NF trait. In contrast, the cold-hardy grandparent *V. riparia* contributed the dominant allele for AD the trait which impact root thickness/diameter. Similarly in tropical and temperate tree species AD correlates with cortical thickness and plays a major role in increasing absorptive capacity and isolating the stele from environmental stress especially in cold climate (Gu et al., 2014; Wang et al., 2019a). Several studies have shown that RSA traits work together to ensure resource uptake and plant survival under environmental stress (Comas et al., 2013; Uga et al., 2013; Koevoets et al., 2016). The SA, TRL, RV, NT, NF, and NL are all strongly correlated and can contribute to increased root system size, which would be beneficial for increased soil exploration, water and nutrient uptake, and strong plant anchorage as shown under drought conditions in *Juglans* and maize (Sun et al., 2011; Comas et al., 2013; Lynch, 2013). In contrast, root diameter impacts cortical thickness and thicker roots support increased absorptive capacity, thus acting as a buffer that protects the stele from environmental stress and contributes to better anchorage (Smart et al., 2002; Aloni et al., 2006; Alvarez-Uria and Körner, 2007; De Dorlodot et al, 2007; Gu et al., 2014; Wang et al., 2019a). The negative correlation between AD (V. *riparia*) contributing the dominant allele and NF (‘Seyval’ contributing dominant allele) provide a point of genetic interaction that may control the overall differences root system morphology in population for the more ‘Seyval’-like and V. riparia-like progeny.

### 4.2 QTL hotspots identify functional genomic units of RSA

Adventitious RSA and soil layer exploration result from an interplay of root traits. Root studies in several grain crops have shown that individual RSA trait QTL co-locate creating hotspots. In 24 rice QTL studies, many QTL for maximum root length co-located creating QTL hotspots (Comas et al., 2013). In *Populus*, 150 QTLs were associated with adventitious root traits and 25 hotspots were identified for these traits (Sun et al., 2019). In a grapevine rootstock population, developed to explore pH related nutrient deficiencies (Bert et al., 2013), 30 QTL for shoot and root traits show root biomass, root section and coarse root number co-localizing in field grown vines using an SSR map (Tandonnet et al., 2018). Similarly, in the VRS-F_2_ population there were seven areas of co-localization encompassing three to seven trait QTLs for chromosomes 1, 9, 13, 18, and 19. Although different traits were phenotyped in field vines with mature root systems, hotspots were identified in similar regions of chromosome 1 and 9 for traits that could contribute to root biomass in either study, providing further confidence in QTL identified in newly rooted cuttings. Genes found to regulate Arabidopsis root development such as auxin signaling were identified in the confidence interval of Tandonnet et al. (2018) and the current study. However, this study identified *LATERAL ORGAN BOUNDARIES DOMAIN* genes (*LBD4* and *ASYMETRIC LEAVES 2*) and *ALFIN-LIKE* transcription factors near the peak positions in the hotspots which have also been shown to play a role in root development. Pathway enrichment was conducted for genes located 700 kb up and downstream of the QTL peak position based on the recommendation that candidate genes be within 3-4 cM of the peak (Cipriani et al., 2011; Hugalde et al., 2021). The AP2_EREBP enriched pathway on chromosomes 1 and 9 included the *ETHYLENE-RESPONSIVE* transcription factor *ERF118*, a gene associated with xylem cell expansion in *Populus* gene co-expression analysis (Seyfferth et al., 2018). In addition, there were two AP2 DOMAIN CONTAINING RAP2 genes that are known to regulate shoot development and are expressed in root vasculature during root development (Che et al., 2006). Another *AP2_EREBP* gene *AINTEGUMENTA* (*ANT1*) often regulating floral organogenesis was found in the chromosome 9 hotspot. This was interesting as a *GROWTH REGULATING FACTOR* (*GRF*) transcription factor gene is a potential target of *ANT1* regulation and this may play a role in the regulation of meristematic competence (Krizek et al., 2020). In *Arabidopsis ANT1* binds to genes similar to another related transcription factor *PLT2* which is known to function in primarily in roots (Krizek et al., 2020). The transport related gene *ALTERED RESPONSE TO GRAVITY 1* (*ARG1*) gene known to interact with *PIN-FORMED* auxin transport proteins regulating the distribution of auxin in response to gravitropic signals is also found in the chromosome 1 hotspot of this study (Konstantinova et al., 2021). In addition, four lateral organ boundary domain genes (*LBD41-3, LBD41-4, LBD21*, and *ASL5*) were found near the peak position for TRL, NT, NF and NL on chromosomes 1 and 13. *LBD* genes are known to have a role in lateral root development in grapevine and other species (Grimplet et al., 2017; Trinh, 2019). The enriched C2C2-CO transcription factors are *CONSTANS-LIKE* (*COL11* and *COL16*) genes known to be involved in floral development. Several *MYB* transcription factor genes with potential roles in root function and development contributed to the enrichment of the *MYB* pathway on chromosomes 1 and 9 (*MYB3R1, MYB62, WEREWOLF-5, MYB7-1, TT2*, and *MYB26*) (Baudry et al., 2004; Gu et al., 2017; Wang et al., 2018; Wang et al., 2019b; Van Den Broeck et al., 2021). *MYB3R1* is a cell cycle related gene that forms a regulatory hub with the *TSO1* transcription factor to coordinate cell division in root and shoot (Wang et al., 2018). *WEREWOLF-5* has a role in regulating root epidermal cell root-hair and nonhair cell types (Wang et al., 2019b). *MYB7* is thought to have a role, in *Arabidopsis* roots, as a repressor regulating *CYC6* a cortex/endodermis asymmetric stem cell division gene (Wang et al., 2019b). Overexpression of *MYB26* in *Populus* promotes secondary wall deposition and is a potential master switch controlling secondary-wall biosynthesis (Xiao et al., 2021). Thus, these genes have the potential to be involved in regulating cell division, root hair and lateral root development. Autophagy, a cytoplasmic degradation pathway, has recently been shown to be involved in glucose-mediated root meristem activity by peroxisome regulation of the production of reactive oxygen species and auxin biosynthesis (Huang et al., 2019; Su et al., 2020). In *Populus*, expression level of several autophagy (*ATG*) genes alternated during the differentiation of xylem and phloem tissues and different *ATG* genes were specific to primary and secondary root tissue development (Wojciechowska et al., 2019). Over-expression of *ATG* in *Arabidopsis* resulted in fewer lateral roots (Su et al., 2020). In the current study, two *AUTOPHAGY* genes (*APG12a* and *ATG18*) were identified underlying the hotspots on chromosome 9 and 13 for NT, NF, and NL traits, suggesting a role in root branching. *ALFIN-like* transcription factors have a role in root hair elongation, meristem development, root development, and abiotic stress and have been shown to enhance drought and salt tolerance (Kayum et al., 2015; Tao et al., 2018). Therefore, the *ALFINDOM1* and *ALFINDOM8* which are identified in SA, RV, TRL, NT, NF, and NL may play a role in the adventitious root morphology. Taken together the enriched suite of transcription factor pathways and the autophagy pathway genes underlying the hotspots for the morphological traits related to root system size and branching patterns present strong candidate genes for further analysis in the genetic control and development of RSA.

### 4.3 Modeling RSA QTL reveals the inter-relationship of small effect traits underlying the RSA complexity

In the present study, all QTL had minor effects that explained less than 10% of phenotypic variance. This is similar to other reports that the RSA is a multigenic trait and often shows 3-6 QTL and a low percentage of variability explanation for individual root traits (Smith, 2010; Bert et al., 2013; Tandonnet et al., 2018). De Dorlodot et al. (2007) suggests that modeling of RSA traits may address the complexity of RSA and reveal interesting relationships between the traits (Comas et al., 2013). They indicate that the possibility of identifying QTL loci in related species using comparative QTL mapping is as an advantage of modeling (Comas et al., 2013). Enriched pathway analysis by trait, in contrast to the analysis of the individual hotspots provided a global perspective of the genes related to each of the root traitsIt is well known that adventitious root initiation is regulated by hormone signaling and a central role for auxin biosynthesis and signaling in lateral root initiation; however, when both the hotspots and modeled QTL were interrogated, several other enriched pathways were identified. Therefore, emphasis here is placed on the less frequently described cell cycle, circadian rhythm, and the *HB* transcription factor family enriched pathways underlying root trait specific QTL (Trinh, 2019).

Unique to modeled traits were distinct cell cycle genes, two *CYCLINA1* and two *CYCLINB1* (CYCA1-1, CYCA1-2, and CYCB1-2) which are expressed in root tips, dividing root cells, lateral roots and root epidermis in *Arabidopsis*, thus also implicating a role in the grapevine RSA (Masucci et al., 1996; Himanen et al., 2002). Clock related genes were found in the enriched circadian rhythm pathway in this study (**Figure 7**, **Supplementary Table 2**). The circadian rhythm pathway had seven genes contributing to its pathway enrichment with five of these having a function in circadian clock signaling (two *EARLY FLOWERING 4 (ELF4), PHYTOCHROME C (PHYC), PHYTOCHROME-ASSOCIATED PROTEIN 1 (PAP1)*, and *ADAGIO PROTEIN 1 (ZTL))* and the signaling gene *CONSTITUTIVELY PHOTOMORPHOGENIC 1 (COP1)*. Light modulates primary root elongation and lateral root development and elongation (Yang and Liu, 2020). In grapevine roots there is a strong network of ABA, cytokinin, and circadian rhythm gene expression during water stress induced growth reduction (Khadka et al., 2019). In roots, the circadian clock is simpler than in the shoot, runs faster in the root tip than shoot, and rephases in roots to controls levels of auxin and auxin related genes during lateral root emergence (Voß et al., 2015; Greenwood et al., 2022). PHYC is active in root tips and has a role in gravitropism in *Arabidopsis* hypocotyls but a limited role in root gravitropism (Tóth et al., 2001; Salisbury et al., 2007; Kumar et al., 2008). ELF4 is a mobile shoot to root signal that promotes regulation of the root clock speed in response to temperature conditions (Chen et al., 2020). The enrichment of the circadian rhythm pathway in several root traits in this study suggest further investigation is warranted as there is limited literature on its function in adventitious RSA. There were six *HB* transcription factors contributing to the enrichment of this pathway for six traits. Of these *PROTODERMAL FACTOR2 (PDF2)* and *ANTHOCYANINLESS2* (*ANL20*) are potential candidate genes in RSA as they play a role in regulating epidermal layer and root cell differentiation and root development (Kubo et al., 1999; Kubo and Hayashi, 2011; Ogawa et al., 2015). These genes regulate cellular organization and the identification of these genes near peak position on chromosomes 13 and 19 support a role in RSA.

## 5 Conclusion

The variation and genetic architecture of own-rooted VRS-F_2_ population are reported. VRS-F_2_ genotypes that had similar root morphology to either grandparent or the parent were identified. ‘Seyval blanc’ contributed to dominant allele for SA, RV, FW, TRL, LR, NT, NF, and NL traits that maximize resource uptake and anchorage. *V. riparia* contributed dominant allele to greater root thickness and link length in the AD, ALSA, and ALL traits which has been noted in species with environmental stress tolerance. QTL hotspots with identical peak positions were identified on chromosomes 1, 9, 13, and 19. These hotspots were underlain by *AUTOPHAGY* genes and *LBD, MYB*, and *ALFINDOM* transcription factor genes that have been shown to have a role in root development in other species. QTL modeling and candidate gene identification revealed interrelatedness of small effect traits causing the RSA complexity. Enriched pathways underline QTL confidence intervals identified genes involved lateral root growth (*LBD* and *PDF*) and cell cycle and circadian clock genes which have previously been shown to have a role in *Arabidopsis* and alfalfa root development. The combined analysis of QTL hotspots and modeled root trait QTLs in a grapevine F_2_ population with grandparents of differing RSA has provided a suite of candidate genes that can be explored for selection of improved adventitious root system architecture in grapevine and other woody species.

## Data availability statement

The datasets presented in this study can be found in online repositories. The names of the repository/repositories and accession number(s) can be found below: https://openprairie.sdstate.edu/, https://openprairie.sdstate.edu/ahps_datasets/1, https://www.try-db.org/TryWeb/Home.php, DOI: 10.17871/TRY.92.

## Author contributions

AF and DA conceptualization, DA acquired data and conducted QTL analysis, AF conducted pathway analysis. DA and AF prepared and finalized the manuscript.

## Supplementary material

Supplementary Table 1 Enriched pathways underlying QTL for identical peak position root traits on chromosomes 1, 9, 13, 19 and the genes contributing to the enrichment.

Supplementary Table 2 Enriched pathways underlying modeled QTL traits and genes contributing to the enrichment.
