# Supplementary figures and images for: Genetic analysis of grapevine root system architecture and loci associated gene networks
Source: Front Plant Sci. 2023 Feb 2;13:1083374. doi: 10.3389/fpls.2022.1083374 (PMC9932984; doi:10.3389/fpls.2022.1083374)

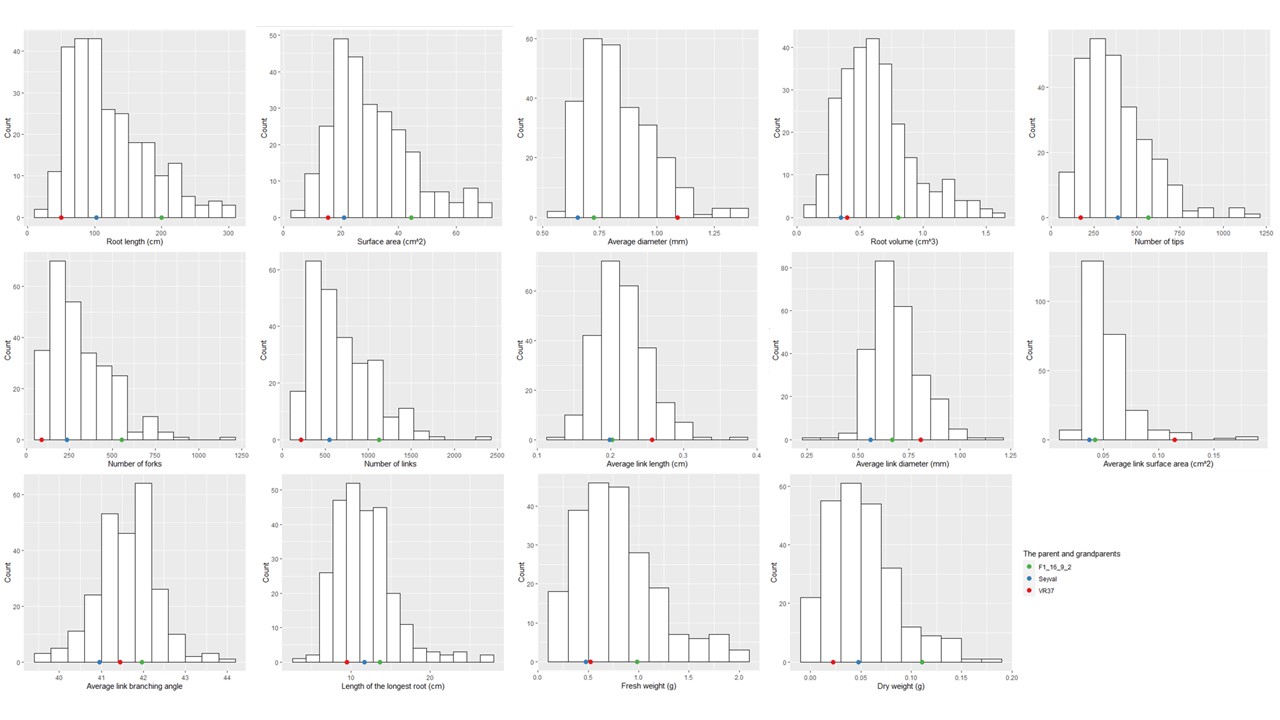

Supplement: Supplementary file 3 [file Image_1.jpeg]
